# Cytotoxic Necrotizing Factors (CNFs)−A Growing Toxin Family

**DOI:** 10.3390/toxins2010116

**Published:** 2011-04-08

**Authors:** Zeynep Knust, Gudula Schmidt

**Affiliations:** Institut für Experimentelle und Klinische Pharmakologie und Toxikologie, Albert-Ludwigs-Universität Freiburg, Albertstr. 25, 79104 Freiburg, Germany; Email: Zeynep.Knust@pharmakol.uni-freiburg.de

**Keywords:** Rho GTPase activation, deamidation, CNF, bacterial toxin

## Abstract

The *Escherichia coli* Cytotoxic Necrotizing Factors, CNF1, CNF2, CNF3 and CNFY from *Yersinia pseudotuberculosis* belong to a family of deamidating toxins. CNFs deamidate glutamine 63/61 in the switch II region of Rho GTPases that is essential for GTP hydrolysing activity. Deamidation leads to constitutive activation of Rho GTPases. However, cellular mechanisms like proteasomal degradation of the activated Rho proteins restrict the action of the GTPases. This review describes the differences between the toxin family members concerning expression, cellular entry and substrate specificity.

## 1. Introduction

Small GTP binding proteins of the Rho family have been identified as common targets of bacterial toxins. The GTPases are involved in the regulation of several cellular processes including re-organization of the cytoskeleton, endo- and exocytosis, cell proliferation and apoptosis (for a review see [1]). They act as “on-off” switches, cycling between an inactive, GDP-bound state and an activated, GTP-bound state, which is recognized by effector molecules. Activation occurs by guanine nucleotide exchange factors (GEFs) that catalyze the release of the bound nucleotide and therefore the exchange of GDP for GTP. Like all small GTPases, Rho proteins return to the GDP-bound state by intrinsic or GTPase activating protein (GAP)-stimulated hydrolysis of GTP. Additional factors regulating Rho GTPase activity and function are GDIs (Guanine nucleotide Dissociation Inhibitors), which sequester GDP-bound Rho proteins in the cytosol or mediate the transport of the activated GTPases to other cellular locations.

The first toxins known to modify Rho GTPases led to complete inactivation of the proteins. C3-like toxins from *Clostridium botulinum*, *Clostridium limosum* and *Staphylococcus aureus* ADP-ribosylate Asn41 of RhoA. This modification enhances the affinity for GDI and thereby inhibits nucleotide exchange, which besides other effects leads to inhibition of RhoA-dependent signalling pathways.

The large clostridial cytotoxins *Clostridium difficile* toxins A and B, which are implicated in antibiotic-associated diarrhea and pseudomembranous colitis, glucosylate RhoA at Thr37. This modification blocks the interaction of the GTPase with effector proteins. Also Rac and Cdc42 are inactivated. Other members of this toxin family like *Clostridium sordellii* lethal toxin possess different substrate specificity and modify Rac but not RhoA. In addition, Ras subfamily proteins (*e.g.*, Ras, Ral and Rap) are modified (for review see [2]). 

The first toxin described to dominantly activate Rho GTPases was the Cytotoxic Necrotizing Factor 1 (CNF1), which is produced by pathogenic *Escherichia coli* strains. It obtained its name from its necrotizing effect on rabbit skin when it was isolated and tested [3]. CNF1 is the best analysed member of the growing toxin family with identical (CNFs) or similar (DNT, Dermonecrotic Toxin) activities described below. 

Uropathogenic *Escherichia coli* (UPEC) are one of the most prevalent strains in infections of the urinary tract [4,5]. It is generally accepted, that most of the UPEC strains live in the intestine and enter the urinary tract via the urethra. While acute urinary tract infections (UTI) can be treated with common antibiotics, the chronic recurring UTIs are a bigger threat. They bear a growing risk of septicaemia due to the invasion of bacteria into the bloodstream. 

CNF1 is a major virulence factor of UPEC strains [6]. It is associated with several other virulence factors like aerobactin, P fimbriae and hemolysin. The gene for hemolysin shows the strongest correlation to *cnf1* in UPEC strains. It was reported, that 98% of *cnf1+* strains were also positive for *hly* [7]. Rippere-Lampe *et al*. could show that *cnf1+* strains exhibit a significantly higher survivability in the UTI mouse model [8]. The infection with *cnf1+* UPEC accompanied with an increased inflammation rate, as it was also reported by Fournout *et al*. [9]. However, recent studies show, that CNF is not only associated with uropathogens. It also is associated with skin and soft tissue infections [10]. Moreover, CNF1 is produced in some extraintestinal *E. coli* (ExPEC)*.* It has been reported for *E. coli* K1, that CNF1 increases the invasion of brain microvascular endothelial cells (BMEC) [11]. This invasion requires cytoskeletal rearrangements due to the activation of RhoA. *E. coli* K1 is one major pathogen participating in neonatal meningitis [5]. In a recent study, it was shown that of 60 patients who died of septicaemia, CNF1 producing *E. coli* were isolated from the blood of 22% of the patients [12]. CNF1 was found in bacteria isolated from meningitis affected children. CNF2 has been demonstrated in *E. coli* isolated in calves and lambs with diarrhoea [13], whereas CNF3 has been identified in Necrotoxigenic *Escherichia coli* (NTEC) from healthy sheep and goats [14]. In contrast to CNF1 and CNF3, which are chromosomally encoded, CNF2 is located on a transmissible plasmid [15]. PCR studies showed that the gene for CNF1 is more common than that for CNF2 [16].

Many studies describe CNF1 as an important virulence factor in bacterial infections: *E. coli* strains, which are CNF1-deficient, have less potential to colonize the urinary tract [17]. Moreover, CNF1 is responsible for tissue damage and disturbs the epithelial barrier function as determined in tissue culture systems [18,19] and affects the function of immune cells by blocking phagocytosis [20]. Induction of phagocytic behaviour of non-phagocytic cells, and inhibition of phagocytosis in monocytes was observed following CNF1 intoxication of cultured cells [17]. There are hints that CNF1 may be involved in cancer development: CNF1 induces the expression of cyclooxigenase-2 (COX-2), activates nuclear factor-kappa B (NF-κB), increases cell motility and inhibits apoptosis [21,22,23].

## 2. Mode of Action

**Figure 1:**
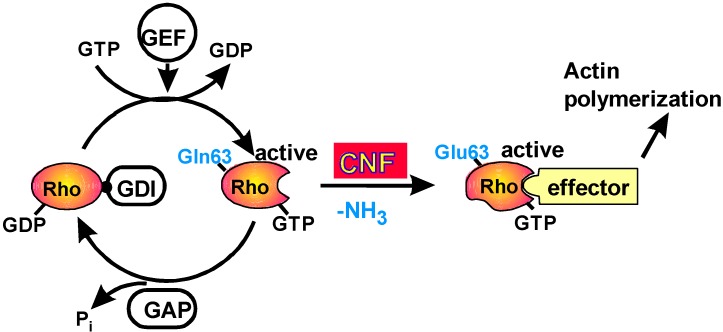
Activation of Rho GTPases by CNFs.

All toxins which activate Rho GTPases by covalent modification modify the same amino acid in Rho GTPases (glutamine 63 in RhoA or its homolog in other Rho proteins is crucial for the GTPase activity). CNFs catalyse the deamidation of this glutamine [24,25]. Therefore, GTP hydrolysis is blocked, which results in continuous effector activation (Figure 1). Meanwhile four CNF isoforms are known: CNF1, CNF2 (85% sequence identity with CNF1) and CNF3 (70% sequence identity with CNF1) from *Escherichia coli* and CNFY (61% sequence identity with CNF1) from *Yersinia pseudotuberculosis*. The Dermonecrotic Toxin (DNT) from *Bordetellaspp.*, which has sequence homology to the CNFs exclusively in the catalytic domain, transamidates glutamine 63 of RhoA [26]. DNT is able to catalyse deamidation of RhoA, but favours transamidation [27,28]. Both modifications lead to inhibition of the intrinsic and GAP-stimulated GTPase activity. However, attachment of a primary amine (*e.g.*, spermidine, putrescine or lysine) to RhoA by transamidation induces a different phenotype on mammalian cells as compared to the phenotype induced by deamidation. This is probably, because the attached amine inhibits some protein-protein interactions [29]. Under certain *in vitro* conditions, CNF1 is able to catalyze transamidation, but primarily, it acts as a deamidase [30]. Unexpectedly, deamidated RhoA shifts to higher molecular weight in SDS-PAGE, although the mass increase due to deamidation is only 1 Da. The same shift can be observed with recombinant proteins (wildtype RhoA and RhoA-Q63E). Obviously the amino acid composition rather than further modifications in the living cell are the reason for the apparent mass increase. In contrast, transamidated RhoA with a much higher mass increase migrates at lower molecular weight [24]. Modification of Rac1 or Cdc42 cannot be monitored by SDS PAGE. 

Due to the constitutive activation of Rho-GTPases, CNF1-intoxication induces characteristic morphological changes in mammalian cells [31]. The most striking differences occur by changes of the actin cytoskeleton. Constitutive activation of RhoA leads to the formation of stress fibers, which are contractile bundles of parallel actin filaments and myosin. Rac activation leads to the formation of lamellipodia, which require a net-like organisation of filamentous actin. The toxins also induce filopodia, finger like structures, due to the activation of Cdc42-dependent pathways. Besides extreme flattening of the cell body, culture cells acquire a multinucleated phenotype. This is considered to simply arise from inhibited cytokinesis with ongoing nuclear division [32]. However, nuclei do not divide equally. Division is asymmetric and aneuploidy is induced [33]. 

CNFs deamidate a single glutamine in the switch II region of Rho GTPases. This glutamine is conserved between different members of the Ras superfamily of small GTPases. However, CNFs exclusively deamidate Rho-proteins. The substrate specificity differs between the CNF isoforms. CNFY is specific for RhoA, B and C and does not modify Rac or Cdc42 [34]. CNF2 is reported to preferentially modify RhoA and Rac [35], whereas CNF1 and CNF3 deamidate RhoA, Rac and Cdc42. However, CNF3 activates RhoA much stronger than CNF1 [36]. 

Although deamidated Rho GTPases do not hydrolyse GTP and therefore constitutive activation is expected, some signalling pathways have been found to be only transiently activated. The reason for this transiency lies in the fate of the modified GTPases. 

Following deamidation by CNF1, cellular Rac was found to be ubiquitinated and subsequently degraded by a proteasome-dependent mechanism [21,37]. Degradation of other Rho GTPases seems to be cell type specific. In HUVEC (human umbilical vein endothelial cells), CNF-activated RhoA and Cdc42 are degraded as well [38], whereas in HeLa cells Rac1 is ubiquitinated and degraded, while other activated GTPases were found to be stable [39]. Since degradation of Rac increases cell motility and leads to enhanced internalization of bacteria, it was speculated that sequential Rho GTPase activation and inactivation limits the inflammatory response and promotes survival of bacteria [21].

## 3. Structure-Function Relationship

All CNFs are identical in length (1013/1014 aa CNFY/CNF1, 2, 3) and comprise a modular structure with an *N*-terminal receptor binding domain. In conjunction with the central translocation domain, it mediates cellular entry (see below).

**Figure 2:**
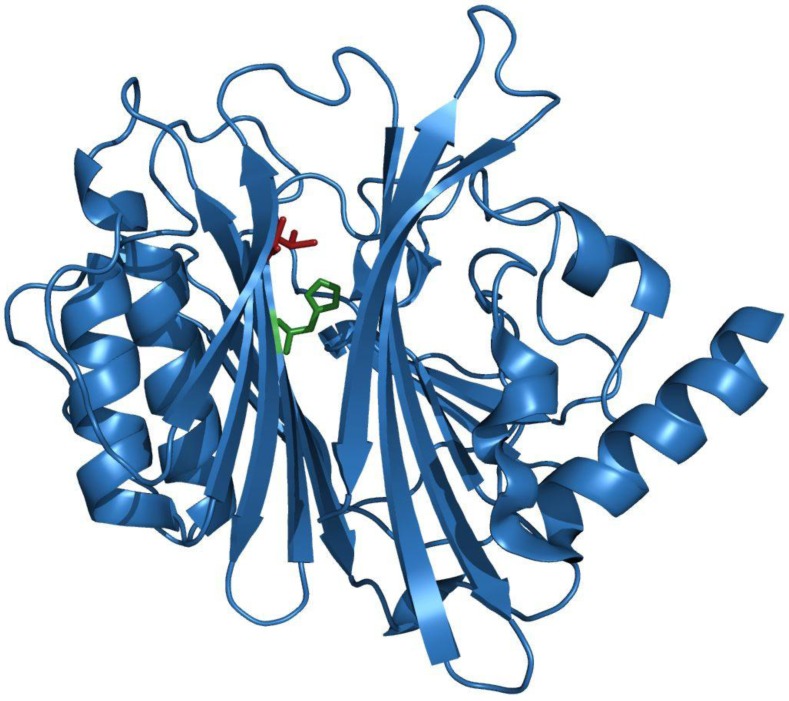
Structure of the CNF1 catalytic domain (aa 710-1014).

The *C*-terminal part of CNF1 (aa 720 to 1014) harbours the full catalytic activity [40,41]. Cysteine 866 and histidine 881 have been found to be crucial for the catalytic activity of CNF1 [41]. The crystal structure identified the third residue of the catalytic triad, valine 833 [42]. Moreover, it revealed a previously unknown protein fold that is considered to be unique to CNFs (Figure 2). Modelling of the CNFY catalytic domain on the CNF1 structure resulted in the same folding [43]. In CNF1 and CNFY the centre of the catalytic domain is formed by a β-sandwich consisting of two mixed β-sheets. It is surrounded by α-helices and nine loop regions that line the entrance to a deep and narrow pocket with the catalytic residues sitting at its base [42].

The catalytic domain is given as ribbon representation. The catalytic residues Cys866 and His881 (side chains shown in red and green, respectively) are located at the base of a deep pocket lined by flexible loops that may be implicated in Rho-binding (Adapted from [42]) using Swiss-PDBViewer 3.7.

The structure presented in Figure 2 suggests that substrate binding occurs by an induced fit mechanism, which is dependent on the flexibility of the switch II region of Rho GTPases. This may be an explanation for the high substrate specificity of the toxins. Buetow and Gosh have defined two of the surrounding loops (loop 8 (aa 964-970) and loop 9 (aa 996-1003)) as important for RhoA recognition by deleting five of the nine loops in CNF1 [44]. Exchange of these loops between CNF isoforms with different substrate specificity (CNF1 and CNFY) allowed modulation of substrate acceptance, which underlines the relevance of the loop regions for substrate recognition and/or binding [43].

### Cellular uptake of CNFs

**Figure 3:**
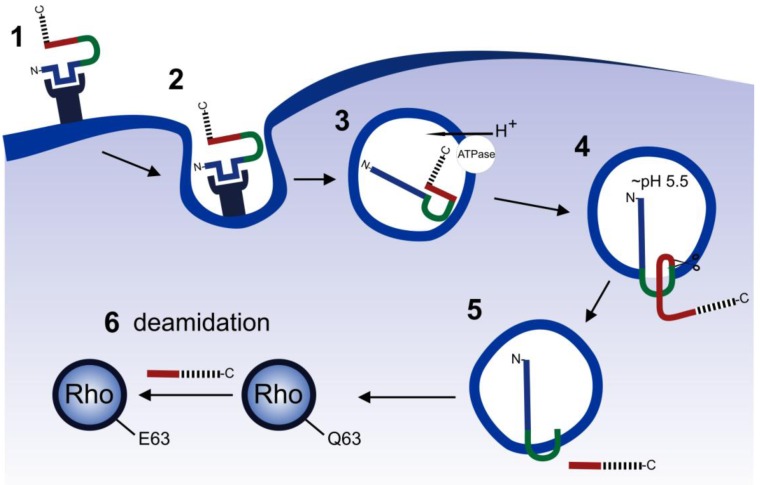
Cellular uptake of CNF1.

The cellular uptake of CNFs is presented in Figure 3. CNF1 enters host cells via receptor-mediated endocytosis [45]. The domain which mediates the interaction to the cell surface ranges from aa 53 to 190 and is able to inhibit the holotoxin activity when given to cells in excess [46]. Using this receptor binding domain as a bait in the yeast two hybrid system, the ubiquitously expressed 37-kDa laminin receptor precursor (37LRP) could be identified as an interaction partner for CNF1 [47]. 37LRP is known to be a precursor protein for the 67-kDa laminin receptor. Binding studies with CNF1 revealed that the 67-kDa laminin receptor clusters with invasive *E. coli* K1. This clustering is due to the expression of CNF1 in these pathogens and promotes their invasion into cells [48]. However, competition studies with CNF1 and CNFY suggest, that CNF1 has a co-receptor in addition to the laminin receptor. Inhibition of heparansulfate proteoglycane (HSPG) expression causes retarded cell entry of CNF1 and no uptake of CNFY [49]. Thus, HSPGs may represent the receptor for CNFY and the co-receptor for CNF1, respectively. This finding is also consistent with the different spectrum of cells, intoxicated by CNF1 and CNFY, respectively. Furthermore, a recent study also suggests a co-receptor for CNF1 due to a second receptor binding part within the CNF1 sequence including the amino acids 683 to 730 [50]. 

The uptake of CNF1 and CNFY into vesicles is independent of clathrin and caveolin [45,49]. Internalized vesicles are acidified by a vacuolar type H^+^-ATPase (v-ATPase) to a pH of 5.5-6.0. Decreasing the early endosomal pH is one of the essential steps in toxin translocation. This has been shown also for many other AB-type toxins. Endosomal acidification can be blocked by bafilomycin A1, a compound from *Streptomyces griseus*, which inhibits the vesicular type proton pumps [51]. The prevention of the endosomal acidification leads to a complete inhibition of CNF activity on cultured cells [45].

The hydropathy profile of CNF1 shows a potential transmembrane domain between the receptor binding and the catalytic domain (aa 300-400). The comparison of the CNF1 primary structure with the one of diphtheria toxin (DT) implies the existence of two short membrane spanning helices H1 (350-372) and H2 (387-412) according to the transmembrane helices TH8 and TH9 in DT [52]. It was postulated, that the hydrophilic loop, which separates H1 and H2, is essential for translocation of the toxin into the host cell cytosol. According to the diphtheria toxin model, the acidic milieu in the endosome leads to a conformational change of the toxin. This forces the exposure of the hydrophobic regions within the translocation domain [53].

Coincidentally, the acidic residues in the loop region are protonated and promote the insertion of helix 1 and helix 2 into the endosomal membrane. According to this model, mutations of acidic amino acids in the loop region result in decreased toxin activity. The charge, as well as the order of these residues in the loop-region are crucial for CNF1 translocation [52] (Figure 3).

Rho GTPases as the targets of CNFs are located in the cytosol and at the plasma membrane. Therefore, the catalytic domain has to be released from the endosomal membrane. Following endocytosis, the catalytic domain of CNF1 is cleaved off. The cleavage site is located between amino acids 536 and 542, a sequence which does not show any sequence similarities to known protease sites [54]. Nevertheless, serine protease inhibitors are able to inhibit the CNF1 deamidating activity as well as its processing. Unlike other AB-toxins, autocatalytic cleavage was not identified, indicating that an endosomal protease may be involved in CNF1 processing. Experiments with CNF1 mutants, which are deficient in the cleavage site, clearly show that the processing and release of the catalytic part from the endosomes is essential for the full biologic activity. It is likely, that this release occurs from late endosomes. Destruction of the microtubules, which are essential for the maturation from early to late endosomes, results in weaker CNF1 toxicity [45,49].

### Export of CNF

Most studies prove the presence of *cnf* in bacteria by PCR. The amount of protein expressed is expected to be very low. This may be the reason, why only little is known about the export of CNFs from bacteria. No typical signal peptide is found in the sequence of CNF1. Two groups show that CNF1 is released from the bacteria to the surrounding environment, tightly associated to outer membrane vesicles. These CNF1-bearing vesicles transfer biologically active CNF1 to mammalian cells [55,56]. Studying the genetic determinants for secretion of CNF1 in meningitis-causing *E. coli*, Yu and Kim suggest that ferredoxin is required for the secretion of CNF across the bacterial inner membrane [57]. However, the exact transport mechanism of CNFs remains to be analyzed.

## 4. Conclusions

Rho GTPases are master regulators of the cytoskeleton and are essentially involved in cell migration, adhesion, polarity, cell division, neuronal plasticity and many other cellular functions. This may be the reason, why Rho GTPases are the targets of various bacterial protein toxins. These toxins inhibit or stimulate Rho proteins and modulate the pattern and ratio of their active and inactive forms in eukaryotic target cells and/or alter the expression and the cellular fate of Rho GTPases. Interestingly, inhibition of Rho proteins seems to mediate the same beneficial effects for bacterial survival as strong activation does. Bacterial protein toxins are meanwhile widely analyzed and are used as important cell biological tools to study the functions of Rho GTPases. Much is known about the cellular entry of CNF1 as a prototype for the family of Rho deamidating toxins. It binds to a cellular protein receptor and is released following endocytosis and cleavage into the cytosol. The molecular action of the CNFs is known and the structure function relationship widely characterized. CNFs constitutively activate Rho GTPases by deamidation of a specific glutamine, required for GTP hydrolysis. The substrate specificity varies between different CNF isoforms. Direct activation of Rho proteins could be of therapeutic relevance and first attempts to use CNF1 for influencing learning and memory or pain have been made [58,59] (summarized in this issue of “Toxins” by Fabbri *et al*.). 
